# How Changes in Extracellular Matrix Mechanics and Gene Expression Variability Might Combine to Drive Cancer Progression

**DOI:** 10.1371/journal.pone.0076122

**Published:** 2013-10-03

**Authors:** Justin Werfel, Silva Krause, Ashley G. Bischof, Robert J. Mannix, Heather Tobin, Yaneer Bar-Yam, Robert M. Bellin, Donald E. Ingber

**Affiliations:** 1 Wyss Institute for Biologically Inspired Engineering, Harvard University, Boston, Massachusetts, United States of America; 2 Vascular Biology Program and Department of Surgery, Boston Children’s Hospital and Harvard Medical School, Boston, Massachusetts, United States of America; 3 Graduate Program in Biophysics, Harvard University, Cambridge, Massachusetts, United States of America; 4 New England Complex Systems Institute, Cambridge, Massachusetts, United States of America; 5 Department of Biology, College of the Holy Cross, Worcester, Massachusetts, United States of America; 6 Harvard School of Engineering and Applied Sciences, Cambridge, Massachusetts, United States of America; Institute for Systems Biology, United States of America

## Abstract

Changes in extracellular matrix (ECM) structure or mechanics can actively drive cancer progression; however, the underlying mechanism remains unknown. Here we explore whether this process could be mediated by changes in cell shape that lead to increases in genetic noise, given that both factors have been independently shown to alter gene expression and induce cell fate switching. We do this using a computer simulation model that explores the impact of physical changes in the tissue microenvironment under conditions in which physical deformation of cells increases gene expression variability among genetically identical cells. The model reveals that cancerous tissue growth can be driven by physical changes in the microenvironment: when increases in cell shape variability due to growth-dependent increases in cell packing density enhance gene expression variation, heterogeneous autonomous growth and further structural disorganization can result, thereby driving cancer progression via positive feedback. The model parameters that led to this prediction are consistent with experimental measurements of mammary tissues that spontaneously undergo cancer progression in transgenic C3(1)-SV40Tag female mice, which exhibit enhanced stiffness of mammary ducts, as well as progressive increases in variability of cell-cell relations and associated cell shape changes. These results demonstrate the potential for physical changes in the tissue microenvironment (e.g., altered ECM mechanics) to induce a cancerous phenotype or accelerate cancer progression in a clonal population through local changes in cell geometry and increased phenotypic variability, even in the absence of gene mutation.

## Introduction

Cancer is commonly thought of as a genetic disease, resulting from a series of gene mutations that deregulate cell growth and lead to neoplastic transformation. While gene mutations contribute to carcinogenesis, recent work has revealed that changes in the tissue microenvironment also can initiate and drive cancer formation. For example, breast cancer formation can be induced in transgenic mice by constitutively expressing a gene that encodes an enzyme that selectively degrades extracellular matrix (ECM) [1], and conversely, some cancer cells can be induced to cease proliferating and differentiate by combining them with normal ECM [2], [3], [4], [5], [6], [7], [8]. Breast cancer progression is also accompanied by progressive increases in ECM stiffness, and breast cancer growth can be selectively accelerated or slowed by respectively increasing or decreasing ECM cross-linking *in vivo* [9], [10]. More recently, breast cancer cells also have been shown to undergo a phenotypic reversion in vitro when physically compressed [11]. But while the importance of the physical nature of the tumor microenvironment is now well appreciated, the mechanism by which these changes might drive (or reverse) cancer formation remains unclear.

We initiated this computational modeling study based on the observation that non-genetic factors also play a critical role in control of cell fate and behavior. One key environmental factor is cell shape [12], which alters gene expression and regulates cell fate switching between growth, differentiation, and apoptosis [13], [14], [15], as well as among different stem cell lineages [16], [17]. Cell shape is, in turn, controlled by changes in mechanical forces that are balanced between the cell’s contractile cytoskeleton and its outside adhesions to ECM and to other cells [13], [14], [15]. Thus, variations in ECM structure or mechanics can alter cell fate switching and influence tissue morphogenesis by altering the force balance between cells and ECM, thereby producing localized cell shape distortion. Moreover, artificially disrupting the cellular force balance by suppressing cytoskeletal tension generation within developing epithelium can lead to disorganized cell-cell relations that mimic those observed during early stages of tumor formation [18]. Thus, it has been suggested that changes in physical interactions between cells and ECM can actively drive or accelerate tumor formation by altering cell shape [19], [20]. But any rise in cell proliferation will be accompanied by an increase in cell packing density that should compress the cells and thereby suppress their growth. And so it remains unclear how changes in ECM mechanics or cell shape distortion could drive cancer formation.

Computer simulations based on dynamic Boolean networks and experimental results indicate that the different cell fates that a particular cell can exhibit (e.g., growth, differentiation, apoptosis) represent a preprogrammed set of common end programs or “attractors” which self-organize within the cell’s regulatory networks [14], [21], [22]. In this type of dynamic network model of information processing, generalized stimuli, such as mechanical forces, and specific molecular cues generate signals which lead cells to follow different trajectories that eventually converge onto one of a small set of common end programs (e.g., growth or differentiation). In addition, because control of cell behavior involves selection of preexisting behavioral modes of the cell, switching also can be induced by genetic noise (i.e., stochastic variations in gene expression profiles). Gene expression stochasticity governs transitions between different fates because while network dynamics driven by specific stimuli tend to drive a cell to a local attractor in state space, transitions between attractors can occur when noise pushes the cell out of one basin of attraction and into another [23]. The environment’s influence on these transitions can be understood as occurring through regulation of the noise amplitude [21], [24]. The importance of these non-genetic factors is emphasized by experiments showing non-genetic variability among clonal cells [25], [26], [27], which reflects stochastic gene expression [25], [26], [28] and responses to different microenvironments [26], [27], [29].

Importantly, morphological loss of regularity of cell shape and position is a hallmark of cancer progression [29], and tumor formation is accompanied by a progressive loss of normal shape-dependent controls over cell growth, differentiation and survival [27], [29], [30], [31], [32]. In addition, the fidelity of genetic control appears to be tightly coupled to nuclear and chromatin structure, which in turn are sensitive to cytoskeletal structure and cell shape regulation [33], [34], [35]. Thus, increases in cell shape variation that accompany early stages of tumor formation could potentially play an active role in cell fate transitions that drive carcinogenesis both by harnessing mechanical signaling pathways and by enhancing genetic variability.

Driven by these considerations, we used a computer simulation model to investigate whether increases in variance in cell morphological parameters caused by changes in ECM structure or mechanics could actively drive cancer progression by increasing genetic noise (gene expression variability) in the altered microenvironment. These modeling studies revealed that deregulation of normal control of cell behavior due to development of structural irregularities in the tissue microenvironment can cause a positive feedback loop that further destabilizes tissue structure, accelerating neoplastic transformation and leading to unconstrained growth. Histological studies in a murine transgenic breast cancer model support our conclusions, and show that cancer progression is associated with progressive increases in the variability of cell shape and cell-cell relations, which our model predicts would promote unconstrained growth.

## Results

### Computational Model of Tissue Homeostasis

The cells and tissues of every organ exhibit characteristic three-dimensional (3D) shapes that are highly regular in form, whereas cell and tissue shape become progressively disorganized during tumor formation and cancer progression. Individual cells also exhibit different behaviors depending on the degree to which they physically extend: in general, anchorage-dependent cells grow more when spread, and they shut off and undergo apoptosis when compact, round or detached from their adhesions, even when cultured in the presence of saturating concentrations of soluble growth factors [13]. The hypothesis we focus on in this work is that irregularities in a cell’s local tissue environment can lead to increased variability in its shape, which may also impair fidelity of cellular genetic control and, hence, lead to increased variability in its responses to the physical forces that act on it to control its growth and viability.

Thus, in our model of tissue form regulation, the phenotypic parameter we focus on is the cell’s behavioral variability, in terms of its propensity to grow in response to physical tension caused by shape distortion. This variability changes as a function of physical factors in the cell’s microenvironment that alter its shape, in particular the number of neighbors it contacts, which changes with cell population density and arrangement. The model is constructed such that cell growth and apoptosis are tightly regulated by forces on the cell (e.g., compaction reliably suppresses growth and increases apoptosis) when a cell is in a “healthy” microenvironment [13], whereas irregularities in the physical microenvironment disrupt that regulation by increasing population variability in cells’ behavioral responses to physical tension or pressure. The aim was to investigate whether these factors alone could result in increases in cell number and tissue mass over time without a genetic mutation occurring in any cell.

Simulation models of carcinogenesis and tumor growth explored in the past have focused at different levels, ranging from individual cells [36], [37], [38], [39] to bulk tissues [40], [41], [42], [43], [44], [45]. Because of the relevant length scales and central importance of variation within cell populations in our study, we chose to construct our simulation as an agent-based model with each agent representing a distinct cell, rather than as a bulk model of continuous tissue. The importance of forces based on relative cell locations dictated an off-lattice [36], [37] rather than cellular automaton (CA) model [38], [46], so that cells can have arbitrary continuous-valued positions rather than being constrained to discrete locations.

**Figure 1:**
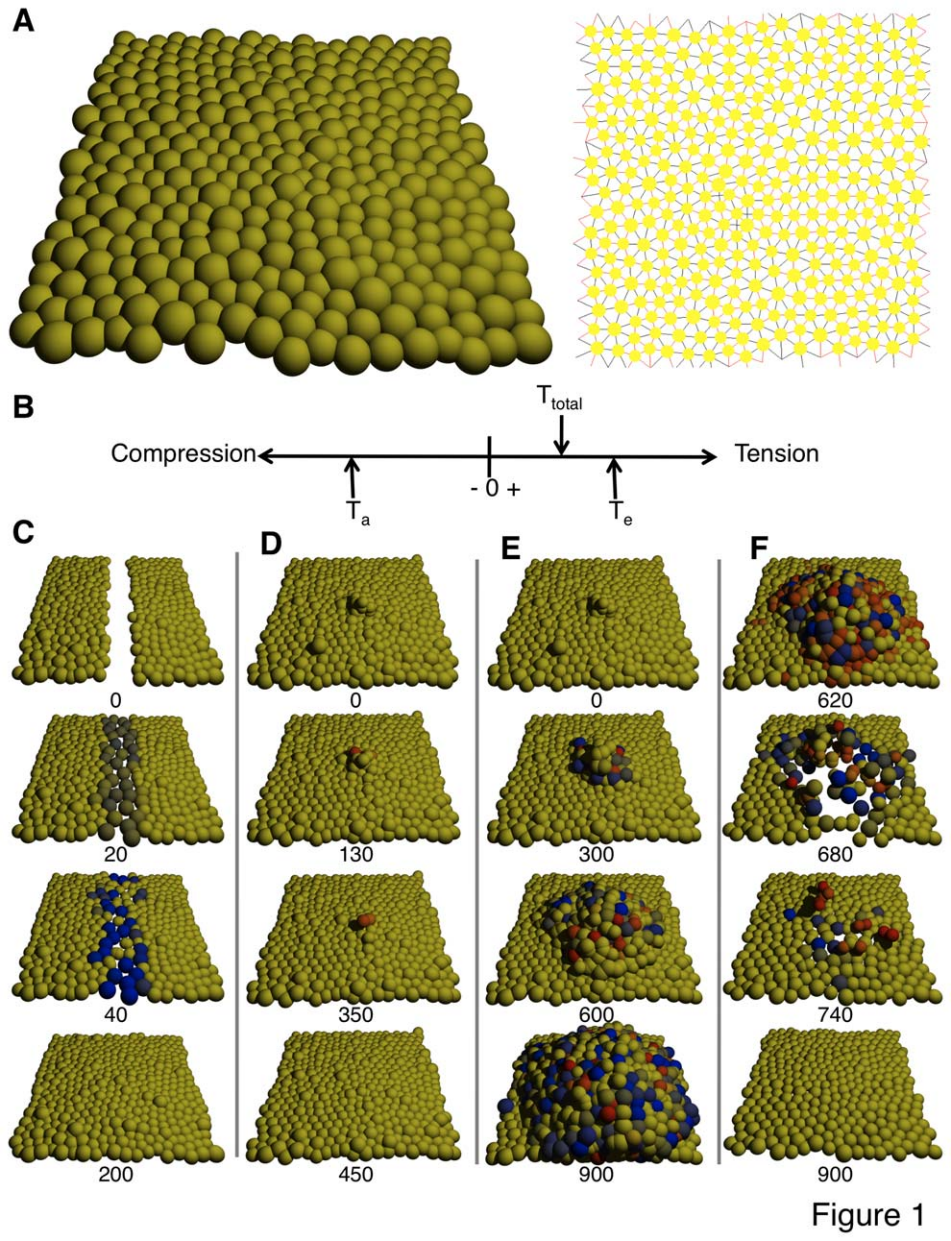
Simulation model demonstrates that behavioral variability in response to microenvironmental irregularity can result in deregulated growth instead of healing. (**A**) Two representations of cells in the model during static equilibrium: (left) space-filling angled view, (right) schematic representation with cells smaller and showing connections between neighbors (black, tension; red, compression). (**B**) The net tension T_total_ on a cell is treated as a scalar value. If the cell is under enough tension that T_total_ exceeds the expansion threshold T_e_, it will gain a volume increment G, dividing after the volume reaches twice its initial value. If the cell is under enough compression that T_total_ falls below the apoptosis threshold T_a_, it will enter apoptosis. (**C**–**F**) Snapshots of healing or deregulated growth processes after perturbation. Numbers below snapshots identify the time step. Animations can be found online as supporting videos. (**C**) Without behavioral variability (σ_e_ = 0), a wound in a monolayer heals quickly as cells proliferate and fill the gap, then cease growth. (**D**) Without variability (σ_e_ = 0), mild initial overgrowth vanishes as cells not in contact with the substrate enter apoptosis. (**E**) With variability (σ_e_ = 2), mild initial overgrowth persists and spreads over time. (**F**) Overgrowth can be reversed and eliminated by applying compressive force to the tissue. Starting at time step 600, cells in the simulation of (**E**) have their value of T_total_ lowered by an amount 2.0. In all panels, cells are tinted blue if they have committed to division (darker as they get closer to the moment of division), red if they have committed to apoptosis (darker as they approach the moment of death), yellow if they have committed to neither fate.

As more than 90% of cancers are epithelial in nature, we constructed our computer simulation model to represent cells in a 3D planar epithelium. In our model, cells act like deformable adhesive spheres on a planar adhesive substrate (Fig. 1A). Each cell experiences a force from each of its neighbors, as though the centers of the two cells were connected by a spring whose rest length is based on the cell sizes. The substrate also exerts forces on cells: vertically to model attachment, and horizontally if a cell borders on an empty area of unoccupied substrate, to model the way a cell spreads in such a case. The net force governs the cell’s movement in 3D space. Outward forces on a cell are registered as tension, inward ones as compression. The net tension on a cell, T_total_, governs its tendency toward growth or apoptosis (Fig. 1B); a cell under greater tension has an increased probability of growth, while greater compression increases the chance of apoptosis [13]. The characteristic level of tension at which a cell tends to switch from quiescence to growth is referred to here as the *expansion threshold* (T_e_). If T_total_ exceeds T_e_ during a time step, the cell adds an increment G to its volume; when the cell reaches twice its initial volume (through reiterative additions of G over time), it commits to division into two cells that each contain the initial volume. If T_total_ drops below the *apoptosis threshold* T_a_, the cell commits to apoptosis. Once a cell has committed to either fate, it waits a further time τ_e_ or τ_a_ and then instantaneously divides or vanishes, respectively.

Increases in ECM stiffness in tissue result in an attachment substrate that more effectively resists cell-generated traction forces (i.e., rather than deforming), and associated mechanical signaling enhances cell contractility [47]; this increases tensile forces exerted on cells for a given geometry. Thus, increasing ECM stiffness corresponds in the model to lowering the values of T_e_ and T_a_, in that it increases cell tension and distortion, which is accompanied by increased cell division and decreased apoptosis for a given geometry [13], [18], [48], [49].

We used the number of cell neighbors as a proxy for detailed cell shape or geometry: a cell in a normal planar monolayer (one cell high) will on average have 6 lateral neighbors, while one particularly crowded or isolated may have significantly more or fewer. When the number of cell neighbors changes in a persistent way, the cell chooses a new value of T_e_ and/or T_a_ from a distribution whose mean is fixed but whose variance (taken to be proportional to a constant σ_e_ or σ_a_, respectively) increases with increasingly irregular neighbor counts (and hence variability of cell shape). Thus, extrinsic factors associated with local neighborhood geometry affect a cell’s entry into proliferation or apoptosis, both through the forces exerted on the cell that trigger those behaviors directly, and through modulation of the cell’s response to those forces. Model details are described fully in the Materials and Methods.

Model simulations revealed that with no population variance in T_e_ (σ_e_ = 0), short-lived disturbances to tissue homeostasis self-heal in that the tissue monolayer morphology returns over time. For example, wounding the epithelium by removing cells within a given area results in wound closure as surviving cells that contact the unoccupied substrate experience forces that cause them to spread out, move into the cleared area, and proliferate until the monolayer is restored after which growth shuts off due to cell compression (Fig. 1C). A hyperplastic epithelium (e.g., induced in the model by adding cells on top of the monolayer) also reverts to a normal monolayer when the abnormal growth stimulus is removed as the overlying cells vanish over time because the tension they experience from their neighbors is too low to support spreading or growth, and both cell rounding and lack of contact with the substrate increase the probability of apoptosis [50] (Fig. 1D). This corresponds to homeostasis in normal living tissues, in which the epithelium maintains its normal architecture when perturbed during wound healing or a temporary increase in growth stimulation through interplay between biomolecular signals and mechanical regulatory cues that alter cell form.

### Microenvironmental Irregularities Result in Increases in Variance of Form Parameters

The computer simulations also revealed that when microenvironmental changes in cell packing result in increased variance (σ_e_>0) in cell expansion behavior (T_e_), it is possible for an otherwise short-lived growth perturbation to persist and spread. For instance, the model predicts that if cell overgrowth occurs and cell piling results for any reason, then persistent unregulated growth can result if there is significant population variance in T_e_ due to creation of an irregular microenvironment that alters the variability of cell shape in the monolayer. Specifically, the increase in cell neighbors in regions of cell piling can lead some of those cells to express abnormally low expansion thresholds, resulting in deregulated growth and disorganization of normal epithelial morphology that is reminiscent of early neoplastic lesions (Fig. 1E). These simulations thus raise the possibility that the emergence of increasing variation in cell-cell contacts, and closely related changes in cell shape parameters caused by any stimulus for cell overgrowth, could feed back to further accelerate the process of tissue disorganization, resulting in cancerous transformation and unregulated cell expansion.

Interestingly, a recent study demonstrated phenotypic reversion of malignant breast epithelial cells to a more normal phenotype when the tissue was subjected to a compressive force [11]. Importantly, our simulation model makes the same prediction: increasing pressure on all cells will preferentially impact those within a tumor-like growth, where cell packing density is higher and pressure is already excessively high; thus increasing pressure will first tend to push these cells away from their expansion threshold, slowing and stopping proliferation. Moreover, still greater pressure leads to the selective death of cells in the tumor as the total pressure crosses their apoptosis threshold T_a_. Remaining cells in the population that retained normal expansion thresholds then repopulate the space left behind, and the tissue remains quiescent with a normal phenotype thereafter (Fig. 1F).

**Figure 2:**
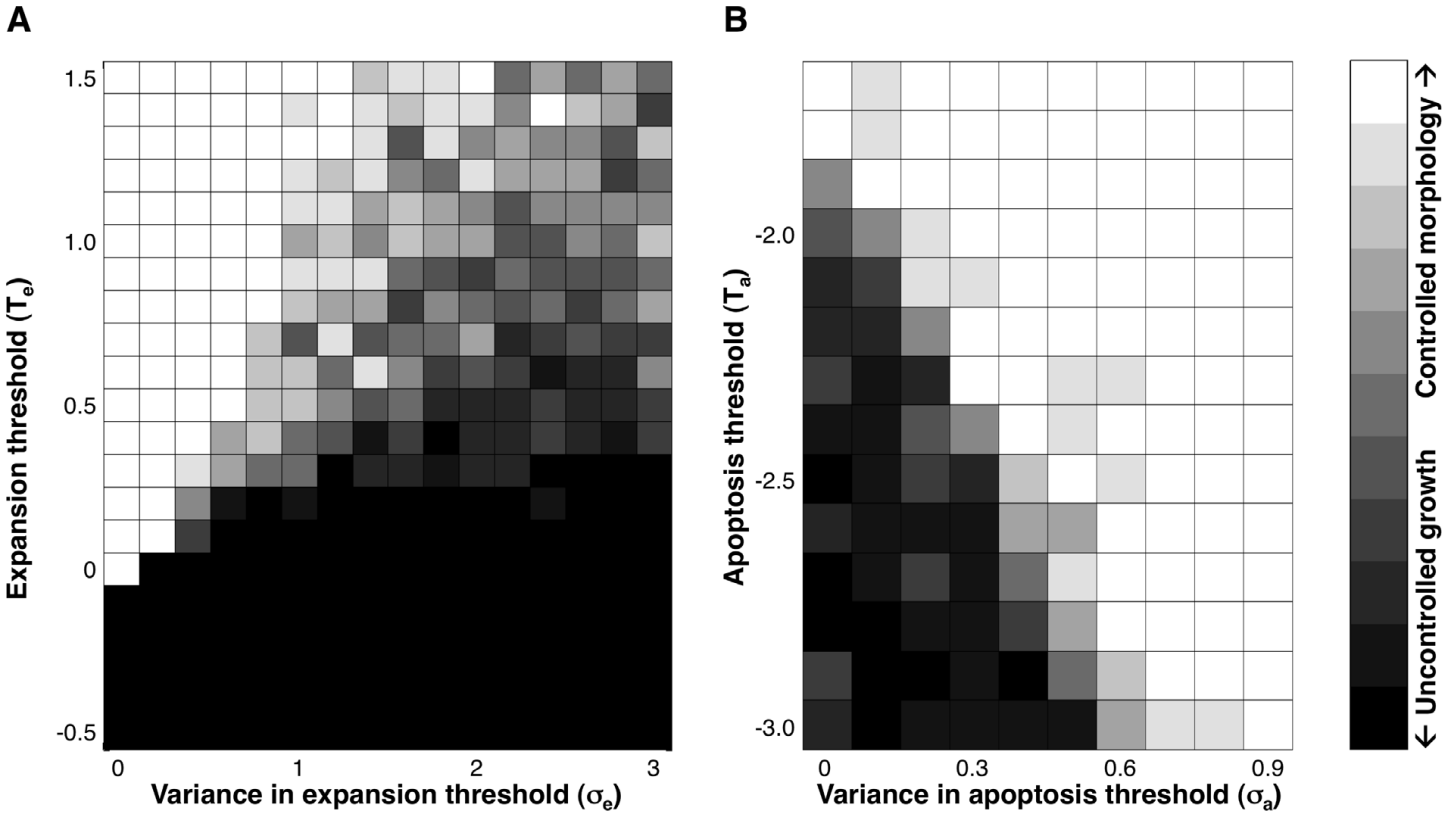
Increasing gene expression variability increases the potential for autonomous growth. For different values of (**A**) T_e_ and σ_e_ or (**B**) T_a_ and σ_a_, pixels show what fraction of ten independent simulation experiments maintain controlled morphology for 2000 time steps, starting from overgrowth (time step 0 in Fig. 1D, 1E): white indicates all runs maintained control, black indicates all resulted in uncontrolled growth, with intermediate pixel values for mixed results. For purposes of these experiments, “controlled morphology” is defined as fewer than 100 cells pushed up out of the monolayer. (**A**) Decreasing T_e_ increases growth and at low enough values can result in continuous growth with no variability (σ_e_ = 0). Increasing variability enables autonomous growth at higher values of T_e_. T_a_ = −3.0, σ_a_ = 0. (**B**) Increasing the value of T_a_ directly or by increasing variability σ_a_ increases cell death in crowded environments, and can prevent unregulated growth due to environmental irregularity from establishing a foothold under conditions where autonomous growth would otherwise occur. T_e_ = 0.3, σ_e_ = 2.0.

It is important to note that decreasing the population’s baseline value of T_e_ in the absence of any population variance (σ_e_ = 0) will increase the equilibrium cell packing density of the monolayer [29], but it will not give rise to uncontrolled growth until T_e_ becomes sufficiently low (Fig. 2A). Increasing variance (σ_e_) enables some cells to exhibit autonomous growth at higher baseline values of T_e_ (Fig. 2A), and thus, preferentially stimulates proliferation in regions of irregular tissue morphology (or altered ECM mechanics). In contrast, the apoptosis threshold T_a_ in the model affects the amount of pressure cells are able to withstand before dying. Microenvironment-related variability in T_a_ can rescue a cancerous phenotype because some cells in anomalously crowded microenvironments express a correspondingly increased probability of apoptosis. This can produce clearing out and normalization of the crowded region, making it more difficult for crowding-linked irregular growth to become established and spread (Fig. 2B). However, variability in T_a_ alone does not give rise to anomalous growth in irregular microenvironments. Thus, we focused on the contribution of modulating the expansion threshold T_e_ in this analysis.

**Figure 3:**
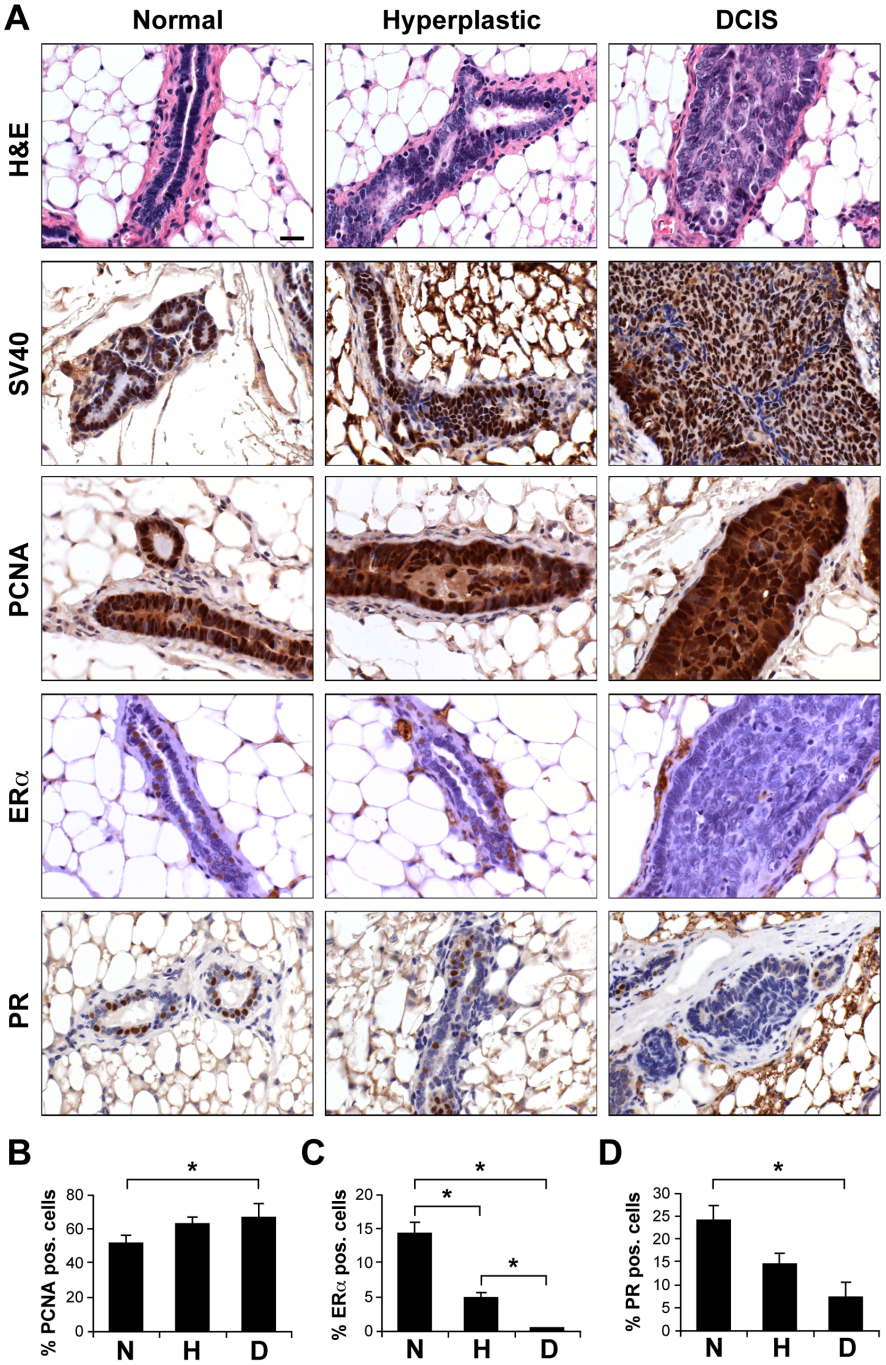
Heterogeneous tumor formation in 16-week-old mammary glands of FVB C3(1)-SV40Tag transgenic mice that contained ducts displaying the morphology of normal epithelium, hyperplastic epithelium, and DCIS lesions. (**A**) Tumor cell proliferation (PCNA) and differentiation (ERα and PR) were altered in hyperplastic and DCIS ducts compared to normal ducts whereas the transgene expression remained similar (SV40). Scale bar: 20 µm. (**B–D**) Morphometric analysis of ductal heterogeneity (N, normal; H, hyperplastic; D, DCIS). (**B**) Epithelial cell growth (% PCNA-positive cells) increased slightly, but significantly in DCIS ducts compared to normal ducts whereas the percentage of cells expressing (**C**) ERα and (**D**) PR decreased significantly in DCIS ducts compared to normal (*, *p*<0.05).

**Figure 4:**
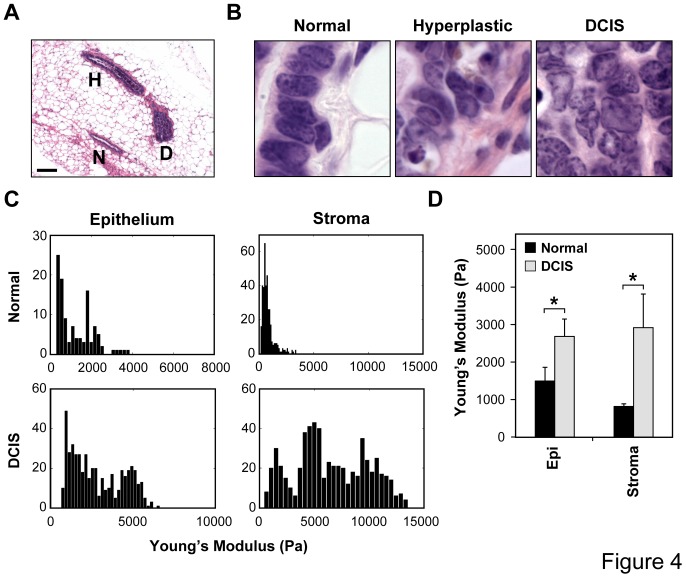
Cell shape and mechanical changes accompany cancer progression in FVB C3(1)-SV40Tag transgenic mice. (**A**) Regional variations in mammary cancer progression observed in the same mammary gland isolated from a 16-week-old transgenic mouse. Note that normal ducts (**N**), hyperplastic ducts (**H**) and DCIS-resembling ducts (**D**) can be found in close proximity in the same gland (scale bar: 100 µm). (**B**) High magnification H&E stainings of normal, hyperplastic and DCIS ducts in 16-week-old transgenic females highlighting epithelial cell shape changes that accompany cancer progression when cells become increasingly pleiomorphic. (**C**) Histograms showing the Young’s moduli of epithelium and periductal stroma of different normal and DCIS ducts measured within the same 16-week-old transgenic mammary glands using AFM. (**D**) Average stiffnesses measured in the epithelial and stromal compartments of normal versus DCIS ducts within the same 16-week-old glands (*, *p*<0.05).

To determine directly whether increases in cell growth in living tissues alter variance in cell shape parameters that are critical for the relevance of our computer model, we analyzed changes in these morphological features during early stages of hyperplasia and formation of ductal carcinoma in situ (DCIS) during breast cancer progression in transgenic C3(1)-SV40Tag mice. Transgenic females spontaneously develop mammary tumors over a time course of 8 to 20 weeks of age in a very robust manner [51], [52]. Cancer progression in these mammary tissues occurs through increased growth and loss of differentiation, as measured by decreased expression of estrogen and progesterone receptors (Figs. 3A–D). The regional heterogeneity of the tissue microenvironment during cancer progression is also clearly evident in this model as individual 16 week mammary glands contain ducts that display different stages of tumor formation (i.e., normal, hyperplastic and DCIS) separated by only small distances within the same gland (Fig. 4A), despite the identical genetic background and similar expression of SV40 large T transgene [51] (Fig. 3A). This ductal heterogeneity was observed in all animals studied and at all time points analyzed.

**Figure 5:**
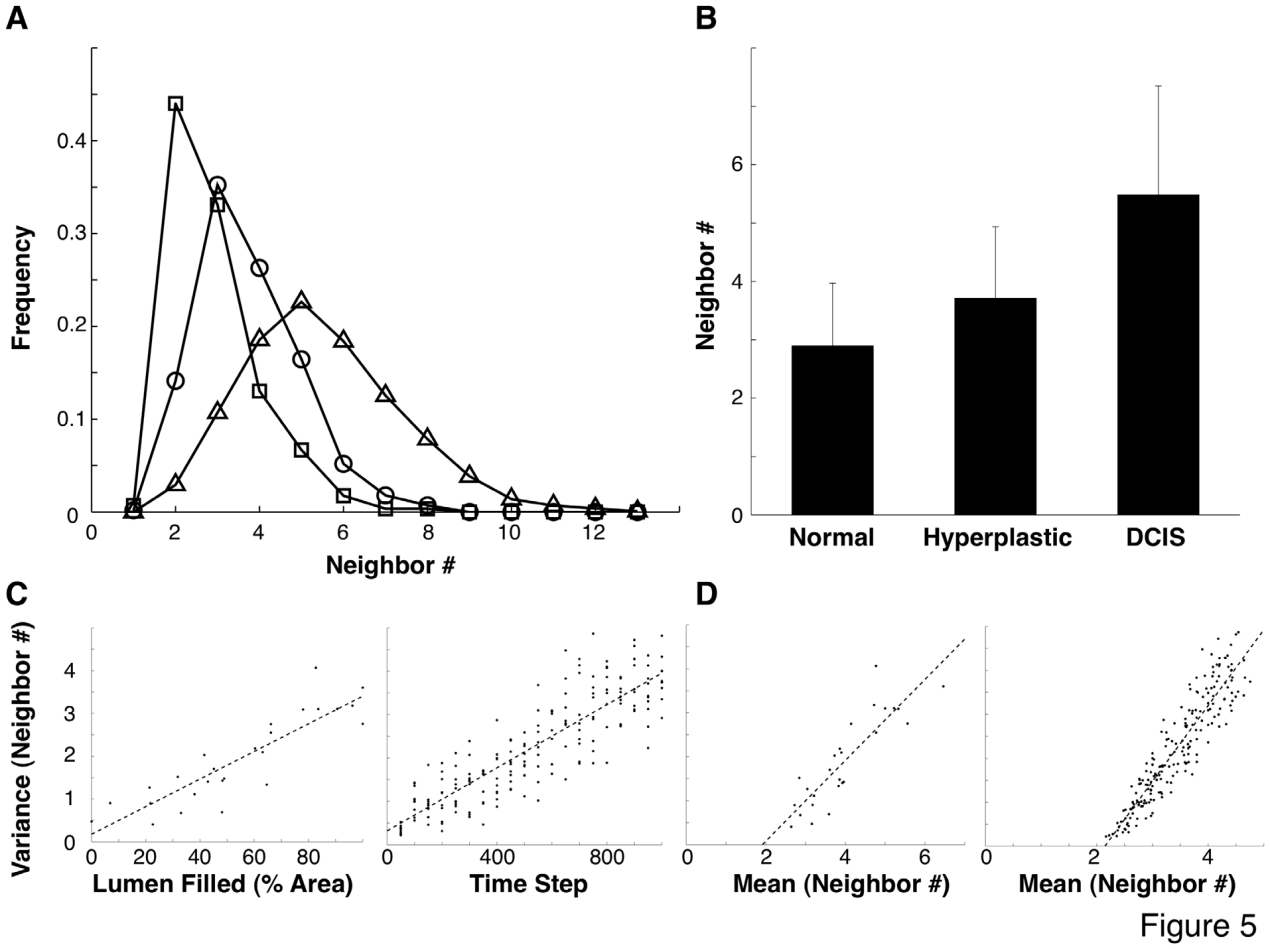
Successive stages in tumor formation are associated with increased mean and variability in cell neighbor count (#). (**A**) Neighbor count frequency for normal (square), hyperplastic (circle), and DCIS (triangle) ducts. (**B**) Mean and standard deviation for neighbor count for each duct class. (**C**) Variance in neighbor count increases with tumor progression, both in histological slices (left, progression measured by duct area filled) and in corresponding slices from simulation model (right, progression measured by time from simulation start). (**D**) Relation between mean and variance in neighbor count in histological slices (left) and corresponding slices from simulation model (right). T_e_ = 0.125, σ_e_ = 0.667.

Importantly, these regional variations in phenotype were accompanied by alterations in cell and nuclear shape (Fig. 4B), as well as local increases in both epithelial and stromal stiffness (Fig. 4C, D). Stiffness was measured using atomic force microscopy (AFM) and both the distribution profile of the stiffnesses measured (Fig. 4C) and the mean stiffness values increased significantly for both epithelium and stroma when normal ducts were compared to DCIS ducts within 16 week glands (Fig. 4D). Cell packing densities increased as well: cells within the epithelial monolayer that lined normal mammary ducts had an average of 2.9±1.1 epithelial cell neighbors (Figs. 5A, B), when analyzed in histological sections using computerized image analysis. Hyperplastic ducts, which are characterized by increased cell proliferation and the presence of cells within the luminal space, showed an increase in the mean number of cell neighbors (3.7±1.2) and analysis of the shape of the distribution also revealed a significant increase in the variance of these data (*p*<0.05; one-tailed F-test; Figs. 5A,B). Cells within the DCIS ducts, which are often enlarged and completely filled by tightly packed cells, showed both the highest mean (5.5) and largest variance (standard deviation = 1.9) in number of cell neighbors, and this level of variance was significantly increased compared to that exhibited by cells in both normal and hyperplastic ducts (*p*<10^−24^; one-tailed F-test). Thus, these results support a key assumption of our simulation model, which is that increased cell growth is associated with a rise in the variance of cell morphological parameters, reflected by variation in the number of cell neighbors, during cancer progression. Interestingly, the increase in variance as the number of cell neighbors increases in the murine model was very similar to that observed in our computer simulation, and similarly the variance in the number of neighbors increased with cancer progression in both the murine and simulation models (Fig. 5).

## Discussion

In this study, we described a simulation model in which increased variability in phenotypic cell parameters, caused by structural variations in tissue microenvironments that alter the number of cell neighbors and cell shape, can lead to sustained growth pathologies that are consistent with cancer progression. No genetic change is necessary and transplanting a misbehaving cell into a normal environment will restore its normal behavior in this model, which is consistent with the observation that some cancerous cells stop dividing and undergo normal histodifferentiation when brought into contact with normal ECM [4], [19], [20], [32], [53] or when physically compressed [11]. Although gene mutations are not required to initiate this process, both decreased genetic fidelity and increased cell proliferation will likely lead to increases in gene mutation rates that could feed back to further accelerate the neoplastic transformation process. This is consistent with the observation that end-stage tumors that result from altering ECM structure alone *in vivo* (e.g., by constitutively expressing the ECM-degrading enzyme, stromelysin) actually exhibit gene mutations and chromosomal abnormalities that are classic hallmarks of malignancy [1].

Although our results were the product of a computer simulation, histological and micromechanical analyses of mammary glands from transgenic C3(1)-SV40Tag mice at 16 weeks of age confirmed that local variations in breast cancer progression that are observed in individual mammary ducts correlate with local changes in mammary tissue mechanics, as well as increased variability in cell shape and cell-cell neighbor relationships. Importantly, these increases in structural variability that associate with tumor progression *in vivo* corresponded closely to the variations in neighbor relationships that we assumed to be affecting behavior in our simulation model (Fig. 5).

Virtually all past work on carcinogenesis has focused on the genetic basis of the disease; however, it is also crucial to understand how non-genetic factors contribute to cancer formation because it is now clear that microenvironmental cues, including local changes in ECM, angiogenesis and the surrounding stroma, can play an equally important role in cancer formation and progression [19], [34], [54], [55]. Most previous computer models of tumorigenesis primarily focused on genetic mutation [43], [56] or on angiogenesis and vascularization [39], [40], [41], [45], often with the goal of making detailed quantitative predictions of growth [38], [43], [46]. In contrast, we considered the impact of physical forces as a growth trigger, and of gene expression variability among genetically identical cells as an enhancer of cancer progression, which to our knowledge has not been explored previously. One of the most important new insights from this analysis is that gene expression variability alone can enable autonomous cell proliferation in an otherwise highly regulated growth environment. Cell shape-dependent growth control of normal tissues, and its progressive loss during neoplastic transformation [29], have been long recognized. However, it has not been possible to explain how autonomous growth might result from these changes in mechanical regulation of cell metabolism because progressive loss of shape sensitivity would generally result in generation of stable cell layers with higher steady-state packing densities, rather than unconstrained proliferation (until complete loss of shape sensitivity is obtained). Our results show that adding environmental variability in the context of the importance of gene expression variation for fate switching provides one explanation for how this switch to autonomy can occur.

The simulation model we considered is considerably simplified, due to our focus on qualitative behavior rather than quantitative precision, with the goal of investigating the feasibility of a previously unexplored mechanism that could contribute to cancer progression. The model includes the elements relevant to the process of interest, and omits or simplifies many biological details, a general necessity for such models. For instance, in common with various past cancer simulations, there is no explicit representation of ECM [36], [39], [44], vasculature [37], [44], angiogenesis [42], [43], [44], or oxygen/nutrient availability [36], [40], [41]; similarly, forces between cells are modeled as springs rather than using representations of more complex deformations of cell shape and cell-cell adhesions [36], [37], [38]. Nevertheless, our results show a general mechanism that does not depend on these detailed elements, and that will likely play an equally important role in more complex models that represent them with greater fidelity.

Our qualitative simulation results were robust across a wide range of parameter values and variation in the details of the model structure. This property is key to the model’s results being of general applicability, and their biological relevance. The specific mean value for T_e_ we used for experiments is close to values for which runaway growth can occur even without variability (Fig. 2). This value was chosen so that the behaviors of interest would reliably occur in the very small section of tissue modeled. We would expect the corresponding value in real cells to be much further from unrestricted growth, given the rarity of such events in tissues composed of billions of cells.

We constructed the simulation model such that a cell placed in a given microenvironment chooses fixed values of T_e_ and T_a_ that do not change until its environment changes; that is, the population variance for the set of cells with a given number of neighbors will be greater for irregular environments than for regular ones, but individual cells do not show stochastic variation while in a fixed environmental condition. An alternate choice would be to increase individual stochasticity in irregular environments, such that cells with an irregular number of neighbors dynamically choose new values of T_e_ and T_a_ over time even without the environment changing. The effect can be the same in a tissue since both choices increase the range of variation in cell properties at any time. Additionally, in a dynamic tissue, the environment changes frequently, so that individual cells in the model as we have constructed it exhibit temporal stochasticity due to environmental fluctuations.

Alternate mechanisms allowing an increased tendency toward growth in irregular microenvironments could produce the same kinds of qualitative results we have demonstrated. For instance, progressively increasing the cell’s tendency for growth (lowering the mean of T_e_ rather than increasing its variance) in response to this disordered microenvironment could similarly lead to unconstrained growth. We focus on variability here, however, because of the evidence for the significance of gene expression variation in clonal populations, and because this could enhance the likelihood for neoplastic transformation even within cells that retain some degree of cell shape sensitivity, as is observed in many transformed cells [29].

The theoretical possibility that altered tissue mechanics could promote cancer progression by altering cell shape and thereby increasing gene expression variability can help to explain multiple clinical observations. For example, it has long been recognized that certain tumors can be triggered by wounding or mechanical trauma, and this is thought to be due to changes in the microenvironment, such as altered ECM dynamics and immune surveillance [57], [58], [59], [60]. But the mechanism by which autonomous growth results in such situations remains unknown. Our simulation shows that cell overgrowth due to wounding or repeated stimulation (e.g., irritants in cigarette smoke, environmental carcinogens, mechanical trauma) can persist and spread, if some cells in the irregular microenvironment respond by enhancing their tendency to grow even when compressed due to gene expression variability.

In summary, our results demonstrate the potential for physical changes in the tissue microenvironment to induce a cancerous phenotype or accelerate cancer progression in a clonal population through local changes in geometry and increased phenotypic variability alone, even in the absence of gene mutations.

## Materials and Methods

### Computational Simulation Model

#### 1. Determining neighbors

The model keeps track of the position in 3D space of each cell’s center and its associated volume, but not its detailed geometry. Instead, a probabilistic cellular automaton (CA) is used to determine which cells are physical neighbors. (Note the distinction between cells in the model and lattice sites in the CA: each cell corresponds to many sites (with the number of sites depending on the cell volume); each site is associated with a single cell, or none at all. The CA is discretized, while cell positions and volumes are real-valued. The CA is used as an auxiliary tool solely to determine neighbor relations.) This approach allows us to find neighbor relations without many of the potential problems of other approaches (e.g., choosing neighbors based solely on distance can link cells that should be physically separated by others; representing cells as Voronoi regions makes volume control difficult; keeping track of cell shape in detail is computationally expensive). The CA discretizes the space into a grid of n_CA_ lattice sites per unit distance. For each cell, the site nearest to its center is designated as belonging to that cell, with other sites initially empty. Empty sites with nonempty sites among their six neighbors are then updated with the identity of one of those nonempty neighbors, chosen at random. This update is performed repeatedly and synchronously (i.e., for all sites at once) until the CA stops changing. Cell size control is handled by limiting the number of sites that can be assigned the identity of each cell, according to the cell’s (discretized) volume. Once the CA has reached a steady state, for any pairs of neighboring sites corresponding to different cells, the two cells in question are designated as neighbors. Similarly, any cells with associated sites that border on the substrate are designated as being in contact with the substrate.

#### 2. Calculating forces

Forces on a cell can have contributions from (1) other cells and (2) the substrate.

1. For each neighboring cell, a force of magnitude kΔr is applied along the line connecting the two, where k is a spring constant and Δr is the difference between the sum of the two cell radii and the distance between their centers. The radius is calculated assuming a spherical cell, r = (3V/4π)^1/3^ for a cell of volume V. In addition to recording the net force F_x_, F_y_, F_z_ on each cell along the three coordinate axes, the net tension/compression T along each axis is recorded as the sum of all component forces directed away from the cell center (e.g., a rightward tensile force due to a neighbor to the right, and a leftward tensile force from a neighbor to the left, both contribute to increased tension along the x-axis; a leftward compressive force due to a close neighbor on the right, and a rightward compressive force from one on the left, both reduce tension along that axis). A single value for each axis records this quantity, which may be designated as either tension or compression according to its sign (positive values are interpreted as tension, and negative as compression).
2. The substrate (taken to be non-movable and non-deformable) exerts a vertical force kΔz on cells in contact with it, where Δz is the cell’s z-coordinate subtracted from its radius. Additionally, the substrate can exert horizontal forces, to model the way that an isolated cell tends to flatten and spread on a substrate. Any lattice sites in the CA with z = 0, that border on empty sites with z = 0, increase both the force and the tension on the associated cell in the direction of the empty site by an amount B.

#### 3. Determining cell behaviors

Each cell’s position at each time step is updated by an amount proportional to the net force on it (with an identity multiplier of 1 distance unit per force unit), up to a maximum distance δ_max_.

The probability that a cell undergoes expansion during a time step, or commits to a path ending in apoptosis, depends on the total tension/compression T_total_ = T_x_+T_y_+T_z_, the cell’s expansion and apoptosis thresholds T_e_ and T_a_, and whether the cell is in contact with the substrate.

For growth, we define
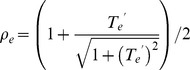
where T′_e_ = (T_total_-T_e_)/s_e_; ρ_e_(T′_e_) is an increasing sigmoid function whose range runs from 0 to 1, with the constant s_e_ affecting the sharpness of the curve (small values correspond to a sharp threshold, while larger values increase the probability of growth under weak tension or lack of growth under strong tension). The probability of growth during a time step is equal to



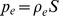
where S = 1 for cells in contact with the substrate and S = S_e_ otherwise. If growth occurs, the cell’s volume increases by an amount G. If the volume reaches twice its initial value, the cell enters a state which will end in division at the end of τ_e_ time steps, during which time further growth or apoptosis will not occur.

Apoptosis is handled analogously. We define
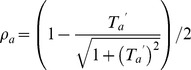
where T′_a_ = (T_total_-T_a_)/s_a_ and ρ_a_(T′_a_) is a decreasing sigmoid function with range from 1 to 0. With probability



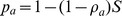
(where S = 1 for cells in contact with the substrate and S = S_a_<1 otherwise–hence a penalty to the probability of survival for cells that lose contact with the substrate (anoikis)), the cell enters a state which will end in apoptosis at the end of τ_a_ time steps, during which time further growth will not occur.

When a cell divides, a new cell is instantaneously created with position equal to that of the mother plus a uniform random value in [−0.05, 0.05] added to each coordinate. The volume of both cells is set to half that of the mother. All other daughter cell parameters are equal to those of the mother.

When a cell undergoes apoptosis, it instantaneously vanishes.

#### 4. Changing cell properties based on neighborhood

When a cell’s microenvironment (in particular, its number of neighbors) changes, it may change its properties such as growth and apoptosis thresholds. Because the probabilistic CA may not produce the same neighborhood relationships at every time step, some form of time averaging is required to prevent changes from occurring due to fluctuations from one time step to the next. Each cell maintains a value n_n_ corresponding to its “true” number of neighbors. If the CA reported the cell as having n_n_ neighbors at fewer than a fraction f_n_ of the last t_n_ time steps, then the true number of neighbors is considered to have changed: n_n_ is set to the number of neighbors reported most frequently in the last t_n_ steps.

When the number of neighbors changes in this way, new values of T_e_ and T_a_ are chosen at random from normal distributions with standard deviations σ_e_Δ_n_, σ_a_Δ_n_ where σ_e_ and σ_a_ are constants and Δ_n_ is the absolute value of the difference between the number of neighbors and the “normal” range of 5 to 8 neighbors. For instance, a cell with 3 or 10 neighbors would choose a new value from a distribution with standard deviation 2σ, while cells with 5 to 8 neighbors will exhibit no variability.

#### 5. Parameter values

As the model is constructed in order to investigate a qualitative phenomenon, rather than to precisely reproduce physiological details with quantitative accuracy, it is important that the qualitative results it produces not depend sensitively on choices of parameter values or on details of the model structure. Varying parameter values changes quantitative results of the model (see, e.g., Fig. 2), but a wide region of parameter space gives intuitively correct qualitative behavior. Parameter values outside this space produce behaviors that are not physiologically relevant in intuitively reasonable ways (e.g., lowering T_e_ enough gives unrestricted growth).

In the experiments reported, unless otherwise specified, we use T_a_ = −3.0, T_e_ = 0.3, σ_e_ = 0.667, σ_a_ = 0, n_CA_ = 5, k = 1.0, B = 0.2, δ_max_ = 0.3, s_e_ = s_a_ = 10^−5^, S_e_ = 1.0, S_a_ = 0.99, G = 0.2, τ_e_ = τ_a_ = 30, t_n_ = 71, f_n_ = 1/4, with initial cell radius of r_0_ = 0.7 units and a 25×25-unit square substrate. These values were chosen based on the qualitative behavior they corresponded to.

### Animals

All animal experimental protocols were approved by the Institutional Animal Care and Use Committee of Children’s Hospital Boston. Experiments presented here utilized a FVB/C3(1)/SV40 T-antigen transgenic mouse model (founder mice were obtained from The Jackson Laboratory, Bar Harbor, ME) and wild type FVB/N mice (Charles River, Wilmington, MA) that were used for breeding and as a control.

### Morphological Studies

Mammary tissues of 16 week old transgenic females were fixed for 16–24 hours in 10% phosphate-buffered formalin (Fisher Scientific, Atlanta, GA), processed and embedded in paraffin. Five micrometer sections were stained with H&E and consecutive sections were used for immunohistochemical analyses. Mouse antibodies to PCNA and PR were obtained from Dako and mouse antibody to ER was purchased from Abcam. An antigen-retrieval method using microwave pretreatment and 0.01 M sodium citrate buffer (pH 6) was used for all antibodies. Images were captured using an AxioCam HR color digital camera attached to a Zeiss Axioscope 2 plus microscope (Carl Zeiss MicroImaging Inc, Thornwood, NY).

For morphometric analysis, 3 to 5 experiments for each condition were analyzed and for each experiment, three arbitrarily chosen fields (20× magnification) were examined per section. Images were captured using a Zeiss Axioscope 2 plus and analyzed with Zeiss Axiovision version 4.8 software. Proliferating epithelial cells were expressed as percent PCNA-positive cells per total epithelial cell number. Computerized quantification using inForm software was used for the measurements of percentage of PCNA (CRi and Caliper Life Sciences, Hopkinton, MA). For all ductal measurements, ducts near the nipple were excluded due to their increased sizes and all ducts measured had cross sectional diameters between 30 and 120 µm. The thickness of periductal stroma and the cross-sectional diameter of ducts were measured using H&E-stained sections. For each ductal phenotype, 8–10 different thickness or diameter measurements were obtained for at least 10–20 different ducts each from at least 3 different animals.

### Atomic Force Microscopy

Mouse mammary glands were embedded in Tissue-Tek O.C.T. freezing medium (Sakura Finetek, Torrance, CA) and sectioned using a cryostat (Leica Microsystems Inc, Buffalo Grove, Il). The 40 µm thick sections were collected on superfrost/plus microscope slides (Fisher Scientific, Atlanta, GA), washed several times in PBS to remove O.C.T. and sections were hydrated in PBS. The stiffness was measured using an MFP-3D-Bio atomic force microscope (Asylum Research, Santa Barbara, CA). Silicon nitride AFM cantilevers with a 60 pN/nm spring constant with either a 5 µm or a 10 µm borosilicate spherical bead on the tip (Novascan) were calibrated thermally according to the Sader method. The tissues were imaged following immunohistochemical staining for laminin 5 and DAPI. The AFM applied a maximum prescribed force of 5–10 nN with an indenter velocity of 2 µm/s. The elastic modulus was determined using the Hertz and the Johnson, Kendall, Roberts (JKR) Model and the Hertz Model was found to be appropriate for this study. To compare average stiffness, several measurements (20–50) of epithelial and stromal stiffness from each gland were averaged and then an average from at least three different animals was calculated and compared.

### Statistical Analysis

SPSS software package 19.0 (SPSS Inc., Chicago, IL) or Microsoft Excel was used for all statistical analyses of mammary gland stainings. ANOVA with a Bonferroni correction were used to compare morphological parameters between the three different ductal phenotypes. Fisher exact probability test was used to compare % proliferating cells using PCNA staining. Independent t-test was used to compare staining intensity and the Young’s Modulus. For all statistical tests, results were considered significant at *p*<0.05. Statistical analyses of PCNA, ER and PR staining are presented as mean ±SEM.

## Supporting Information

Video S1 **Animation of the process shown in** **Fig. 1C** **.**
(MPEG)

Video S2 **Animation of the process shown in** **Fig. 1D** **.**
(MPEG)

Video S3 **Animation of the process shown in** **Fig. 1E** **.**
(MPEG)

Video S4 **Animation of the process shown in** **Fig. 1F** **.**
(MPEG)
